# Novel Zinc-Related Differentially Methylated Regions in Leukocytes of Women With and Without Obesity

**DOI:** 10.3389/fnut.2022.785281

**Published:** 2022-03-07

**Authors:** Natália Yumi Noronha, Mariana Barato, Chanachai Sae-Lee, Marcela Augusta de Souza Pinhel, Lígia Moriguchi Watanabe, Vanessa Aparecida Batista Pereira, Guilherme da Silva Rodrigues, Déborah Araújo Morais, Wellington Tavares de Sousa, Vanessa Cristina de Oliveira Souza, Jessica Rodrigues Plaça, Wilson Salgado, Fernando Barbosa, Torsten Plösch, Carla Barbosa Nonino

**Affiliations:** ^1^Department of Internal Medicine, Ribeirao Preto Medical School, University of São Paulo, São Paulo, Brazil; ^2^Department of Molecular Biology, São José do Rio Preto Medical School, São Paulo, Brazil; ^3^Research Division, Faculty of Medicine, Siriraj Hospital, Mahidol University, Bangkok, Thailand; ^4^Department of Health Sciences, Ribeirão Preto Medical School, University of São Paulo, São Paulo, Brazil; ^5^Department of Clinical Analysis, Toxicology and Food Sciences, School of Pharmaceutical Sciences of Ribeirão Preto, University of São Paulo, São Paulo, Brazil; ^6^National Institute of Science and Technology in Stem Cell and Cell Therapy and Center for Cell-Based Therapy, São Paulo, Brazil; ^7^Department of Surgery and Anatomy, Ribeirao Preto Medical School, SãoPaulo, Brazil; ^8^Department of Obstetrics and Gynecology, University Medical Center Groningen, University of Groningen, Groningen, Netherlands

**Keywords:** zinc deficiency, DNA methylation, age acceleration, epigenetic markers, *PM20D1*

## Abstract

**Introduction:**

Nutriepigenetic markers are predictive responses associated with changes in “surrounding” environmental conditions of humans, which may influence metabolic diseases. Although rich in calories, Western diets could be linked with the deficiency of micronutrients, resulting in the downstream of epigenetic and metabolic effects and consequently in obesity. Zinc (Zn) is an essential nutrient associated with distinct biological roles in human health. Despite the importance of Zn in metabolic processes, little is known about the relationship between Zn and epigenetic. Thus, the present study aimed to identify the epigenetic variables associated with Zn daily ingestion (ZnDI) and serum Zinc (ZnS) levels in women with and without obesity.

**Materials and Methods:**

This is a case-control, non-randomized, single-center study conducted with 21 women allocated into two groups: control group (CG), composed of 11 women without obesity, and study group (SG), composed of 10 women with obesity. Anthropometric measurements, ZnDI, and ZnS levels were evaluated. Also, leukocyte DNA was extracted for DNA methylation analysis using 450 k Illumina BeadChips. The epigenetic clock was calculated by Horvath method. The chip analysis methylation pipeline (ChAMP) package selected the differentially methylated regions (DMRs).

**Results:**

The SG had lower ZnS levels than the CG. Moreover, in SG, the ZnS levels were negatively associated with the epigenetic age acceleration. The DMR analysis revealed 37 DMRs associated with ZnDI and ZnS levels. The DMR of *PM20D1* gene was commonly associated with ZnDI and ZnS levels and was hypomethylated in the SG.

**Conclusion:**

Our findings provide new information on Zn's modulation of DNA methylation patterns and bring new perspectives for understanding the nutriepigenetic mechanisms in obesity.

## Introduction

Nutriepigenetic markers are predictive responses associated with changes in “surrounding” environmental conditions of humans, influencing metabolic diseases. DNA methylation, the most studied epigenetic modification, is defined as a reversible chemical modification (addition of a methyl group) in a CpG dinucleotide, represented as methylated CpG (mCpG) (1). The available studies in DNA methylation typically retrieve a list of trait-associated differentially methylated positions (DMPs) or differentially methylated regions (DMRs) (2). DMPs refer to a single CpG site analyzed, while DMRs refer to a genomic region that comprises several CpG sites. Evaluation of trait-related DMRs can avoid stochastic associations and brings more information about the gene-specific methylation status (3). Another application of DNA methylation profiles is the development of aging epigenetic biomarkers, the epigenetic clock, or DNA methylation age (DNAm age) (4). DNAm age model was developed by Horvath and is based on DNA methylation at 353 CpG sites, which map genes associated with cell development and survival (5). Additionally, Horvath's (5) epigenetic clock is reliable and highly correlated with chronological age. DNAm age acceleration is estimated by the residuals of the regression of chronological age and has been correlated with mortality and aging-related diseases (6, 7). Recent investigations have shown that metabolic diseases, such as obesity, could be associated with changes in DNAm age (8).

Obesity is characterized by an abnormal fat accumulation that leads to other comorbidities and health risks (9). It is a worldwide health concern with a multifactorial etiology, including environmental and genetic influences (9). Eating behavior could be an environmental risk factor for obesity progression (9). Although characterized by excessive high-energy density foods and palatable ingredients, Western diets may result in a high risk for micronutrient deficiency (10–12). Also, Western diets have been described as possible epigenetic modulators due to their inflammatory potential. However, evaluating responses to single nutrients or isolated compounds in epigenome studies remains a challenge (1).

Zinc (Zn) deficiency is common in obesity conditions, resulting in an inflammatory status, oxidative stress, insulin resistance, and lower insulin secretion by the pancreatic β cells (13). Zn participates in DNA repair and transcription, as an enzyme cofactor, and regulates cell proliferation, including apoptosis and cell differentiation (14). Zn deficiency can also be associated with DNA hypomethylation through the methionine synthetase (MTR) and reduction in the activity of DNA methyltransferases (DNMTs). Another Zn-related epigenetic mechanism is its role in maintaining the three-dimensional structure of Zinc finger domains (ZnDs). Recently it was described that these motifs could have a new genomic influence, acting as methyl-CpG binding proteins (MBPs). Those proteins can recognize and bind methylated DNA and promote downstream effects of gene expression (15). Nevertheless, the obesity-related biomarker, peroxisome proliferator-activated receptor-gamma (PPARγ), is a nuclear receptor protein that regulates fatty acid and glucose homeostasis. Zn supplementation may increase PPARγ expression, resulting from the two ZnDs that can bind to DNA (16).

Thus, the purpose of this study was to identify epigenetic variables related to serum Zn (ZnS) levels and Zn daily ingestion (ZnDI) in a case-control cohort of women with and without obesity. Here, we hypothesized that Zn is related to DNA methylation, and this association may have metabolic effects.

## Materials and Methods

### Ethics Statement

The Research Ethics Committee of the Ribeirão Preto Medical School University Hospital of University of São Paulo (HCRP-USP) approved the study with the CAAE license: 14275319.7.0000.5440. All participants signed the informed consent form. The principles of the Declaration of Helsinki guided all the procedures that were adopted (17).

### Study Design

A case-control, non-randomized, single-center study was conducted in Ribeirão Preto Medical School University Hospital (HCRP-USP).

### Subjects

The population size was defined by convenience. Individuals (*n* = 21) were selected and classified according to their body mass index (BMI), following the international classification from the Centers for Disease Control and Prevention (CDC) (18). Obesity is defined by BMI > 30 kg/m^2^ and non-obesity by 18.5 < BMI < 25 kg/m^2^. The volunteers were allocated into two groups: control group (CG), composed of 11 women without obesity, and study group (SG), composed of 10 women with obesity. The inclusion criteria adopted in this study were women aged 18–50 years, with stable body weight for at least 6 months. The non-inclusion criteria were current tobacco use, pregnant women, or women in the post-menopause stage. Patients under any nutritional, surgical, or healthy interventions protocols were not included, and participants had to report any other health condition besides obesity.

### Anthropometric and Body Composition Data

Weight was measured with an electronic platform (Filizola) scale with a precision of 0.1 kg and a maximum capacity of 300 kg. Height was measured with a vertical shaft with 0.5 cm graduation. BMI was calculated by dividing the body mass in kilograms by the square of the body height in meters, universally expressed in units of kg/m^2^. Body composition was measured using the electrical bioimpedance device model Quantum BIA 450 Q—RJL System (Clinton Township, MI, USA). For waist circumference (WC) measurement (cm), the smallest perimeter between the last rib and the iliac crest was adopted.

### ZnDI Evaluation

ZnDI was assessed based on food record over 3 non-consecutive days. Orientation was conducted for the patients to self-record the type and amount of food and beverages consumed. The information was processed in the nutritional analysis program Dietpro® 5i.

### Biological Sampling

Patients were instructed to attend the hospital in the morning after a 12 h fasting period. Serum and EDTA vacuum tubes were used to collect peripheral blood for biochemical, ZnS levels, and genetic analysis. The tubes were immediately centrifuged, and samples were processed and stored at -80°C.

### Biochemical Analysis

The biochemical biomarkers that were accessed included total cholesterol (TC), triglycerides (TG), high-density lipoprotein cholesterol (HDL-c), low-density lipoprotein cholesterol (LDL-c), and blood glucose.

### ZnS Measurements

ZnS levels were determined with an inductively coupled plasma mass spectrometer (PerkinElmer, NexION 2000 B, Waltham, MA, USA, EUA). The analysis was performed using the procedure described by Batista et al. (19). Briefly, the samples (0.25 ml) were directly diluted in a metal-free polypropylene tube containing 4.75 ml of the diluent solution composed of 0.005% v/v Triton® X-100 and 0.5% v/v distilled HNO_3_. The resulting solution was directly injected for inductively coupled plasma–mass spectrometry (ICP-MS) measurements. The analytical calibration standards (matrix-matching) were prepared daily over the range of 10–100 μgL^−1^.

### DNA Extraction and 450K DNA Methylation Illumina BeadChip

Leukocyte DNA extraction was performed using the automated Promega system (Madison, Wisconsin, USA). All instructions stipulated by the manufacturer were followed. The DNA integrity was accessed by agarose gel and the quality, using a nanodrop (Thermo Fisher, Waltham, Massachusetts, USA). Following the recommendations of the Infinium Human Methylation 450k (IHM450K) protocol, DNA was extracted from total leukocytes and converted to bisulfite using the EZ DNA Methylation Kit (Zymo Research Corporation, Irvine, CA, USA). Subsequently, the processes were performed using 500 ng of DNA hybridized to the IHM450K BeadChip. The intensities were obtained by the iScan system (Illumina, Inc., San Diego, CA, USA), and the raw data file was exported for bioinformatics analysis.

### Bioinformatics Analysis

The analysis was performed using RStudio version 3.6.2 and the ChAMP package version 2.24.0, available from Bioconductor (2). We evaluated the anthropometric, biochemical, epigenetic, and Zn information for further association.

After data normalization using the champ.norm() function available in the ChAMP package, we performed a quality control analysis. Missing data were imputed with K-nearest neighbor (k-NN) method (20). Single value decomposition (SVD) function was used to verify the components inserted in the sample sheet and the main variations between methylation data.

The method proposed by Houseman (21) was used to estimate the cell type of leukocytes. The information about the leukocyte cell types was obtained from purified cells, and the IHM450K contained 473 CpG sites as a reference for the most critical cell types.

### Epigenetic Clock Analysis

Using the beta values obtained from the beta-mixture quantile (BMIQ) normalization, the DNAm age was calculated using the Horvath's method through the online tool (http://dnamage.genetics.ucla.edu/) (5). Briefly, the calculation of DNAm age is based on methylation levels of 353 CpG sites. Age-acceleration metrics were derived by regressing DNAm age on chronological age and predicting the residuals (AAR). A positive or accelerated epigenetic age occurred when an individual's DNA methylation predicted age is older than chronological age.

### Differentially Methylated Regions

The champ.DMR() function was used to calculate the DMR. A supervised method, the Bumphunter, was adopted in the DMR calculation. This method is based on linear regression models and uses statistical techniques to scan the genome for groups of probes associated with the variable of interest. A linear regression of the M value is performed to identify candidate regions, and only results with significant false discovery rate (FDR) were exported (argument cutoff point available in the Bumphunter function) (22). M-value, a method proposed to measure the methylation level, is the log2 ratio of the intensities of methylated probe versus unmethylated probe (23). The correspondent symbols were input in the STRING (Protein-Protein Interaction Networks Functional Enrichment Analysis) for gene ontology results (24).

### Statistical Analysis

The anthropometric, biochemical, ZnDI, and ZnS variables were analyzed using the Statistical Package for Social Sciences (SPSS) software version 24.0 (IBM, New York, USA). The Shapiro Wilk test was used to verify the normality of data. Student *t*-test was performed to compare parametric data, and Mann–Whitney U test was used to compare non-parametric data. Spearman's correlation was performed to verify associations between AAR and ZnS levels, BMI and WC. The *p*-value was established at *p* < 0.05.

## Results

### Clinical Data

Table 1 summarizes anthropometry, body composition, and biochemical analysis. As expected for these variables, we found the weight, BMI, fat mass, free fat mass, and WC values to be higher in SG when compared to CG. No difference was found between SC and CG for glucose, TC, HDL-c, LDL-c, TG, and ZnDI values. SG had lower ZnS levels (1.05 ± 0.20 mg/l) compared to CG (1.40 ± 0.21 mg/l, *p* = 0.001).

**Table 1:** Descriptive characteristics of the study population (*n* = 21).

	**SG *n* = 10**	**CG *n* = 11**	***p*** **-value**	**Min**.	**Max**.	**Reference value**
**Variables**	**M ±SD**	**M ±SD**				
Age (years)	37.83 ± 12.05	37.81 ± 11.43	0.997	20	56	-
Weight (kg)	133.75 ± 32.95	58.34 ± 6.92	**<0.001**	47.8	200.8	**-**
BMI (kg/m^2^)	49.65 ± 6.99	22.88 ± 1.90	**<0.001**	18.5	63	BMI > 40-Obese grade III18.5 < BMI < 25-Normal weight (18)
WC (cm)	129.88 ± 13.85	82.94 ± 7.98	**<0.001**	69.9	153	>88.9 cm-Risk (18)
FM (%)	48.83 ± 3.01	31.26 ± 4.87	**<0.001**	25.3	53	20–30% (25)
Glucose (mg/dL)	93.70 ± 12.18	90.33 ± 6.38	0.544	80	116	80–130 mg/dL (26)
TC (mg/dL)	183.30 ± 19.61	200.00 ± 30.95	0.204	142	221	<200 mg/dL (27)
HDL-c (mg/dL)	44.70 ± 8.00	55.00 ± 15.09	0.093	39	84	>60 mg/dL (27)
LDL-c (mg/dL)	118.4 ± 22.84	130.50 ± 25.65	0.343	86	156	<100 mg/dL (27)
TG (mg/dL)	115.56 ± 47.90	72.17 ± 32.94	0.076	71	198	<150 mg/dL (27)
ZnS levels (μg/mL)	1.05 ± 0.20	1.40 ± 0.21	**0.001**	0.56	1.72	0.7–1.20 (28)
ZnDI (mg/day)	9.967 ± 4.55	6.78 ± 2.66	0.062	3.58	15.46	8 mg/day (29)

*Student's t-test for independent samples (p < 0.05); the Cohen's d test varied between 0.5 and 0.8, suggesting a medium to large effect size. n, sample size; M ± SD, mean ± standard deviation; CG, control group; SG, study group; cm, centimeter; mg, milligram; μm, microgram; dL, deciliter; %, percentage; BMI, Body Mass Index; WC, waist circumference; FM, Fat Mass; TC, Total Cholesterol; HDL-c, High-Density Lipoprotein cholesterol fraction. Cholesterol; ZnS, Serum zinc; ZnDI, Zinc daily ingestion; LDL-c, Low-Density Lipoprotein cholesterol fraction; TG, triglycerides. p-value < 0.05*.

### Epigenetic Clock Analysis

**Figure 1:**
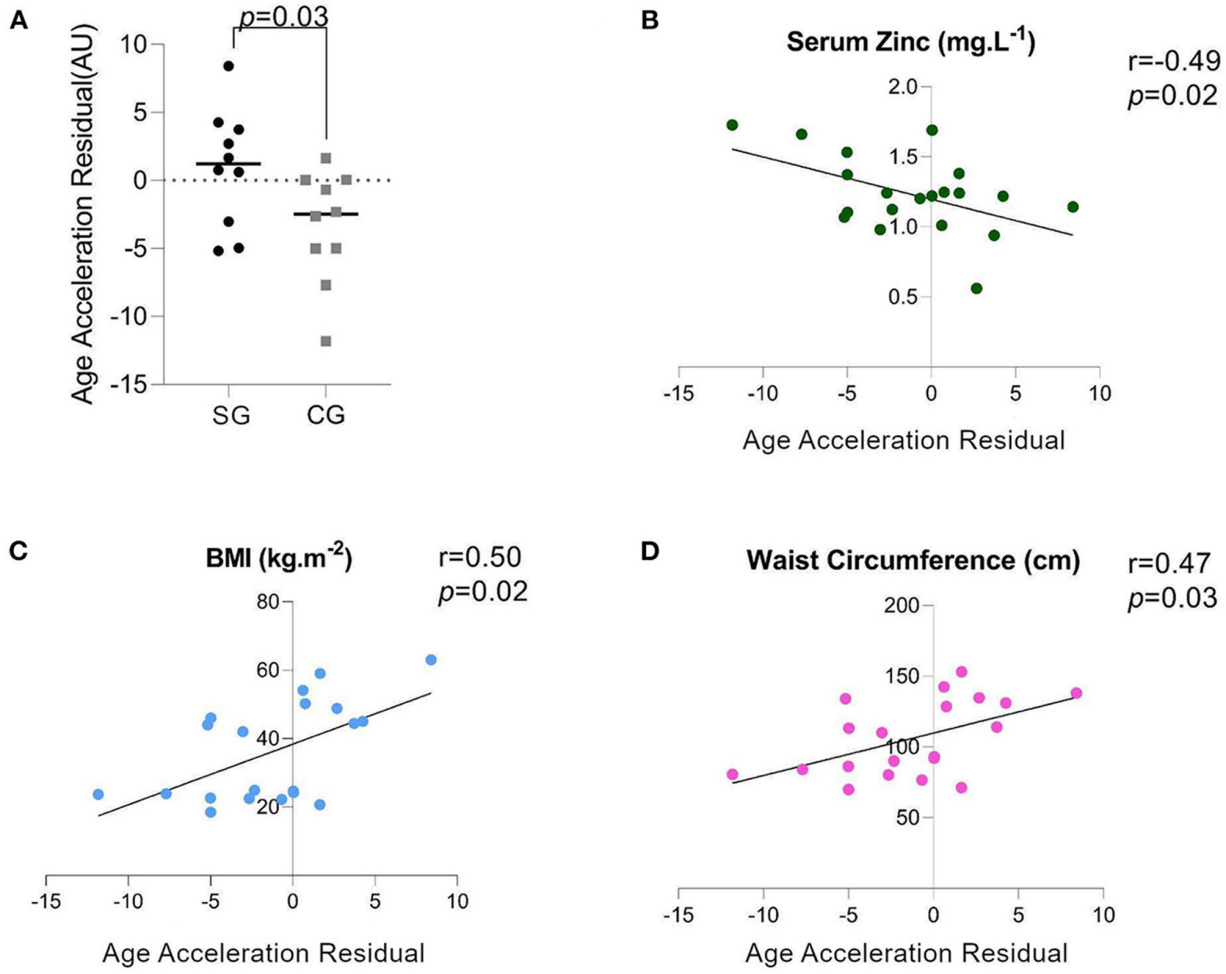
**(A)** AAR: Age Acceleration Residual (measured in arbitrary units), **(B)** Spearman's Correlation of the serum zinc (ZnS) levels and the AAR, **(C)** Spearman's Correlation of the BMI and AAR, **(D)** Spearman's Correlation of the waist circumference (WC) and AAR.

**Figure 2:**
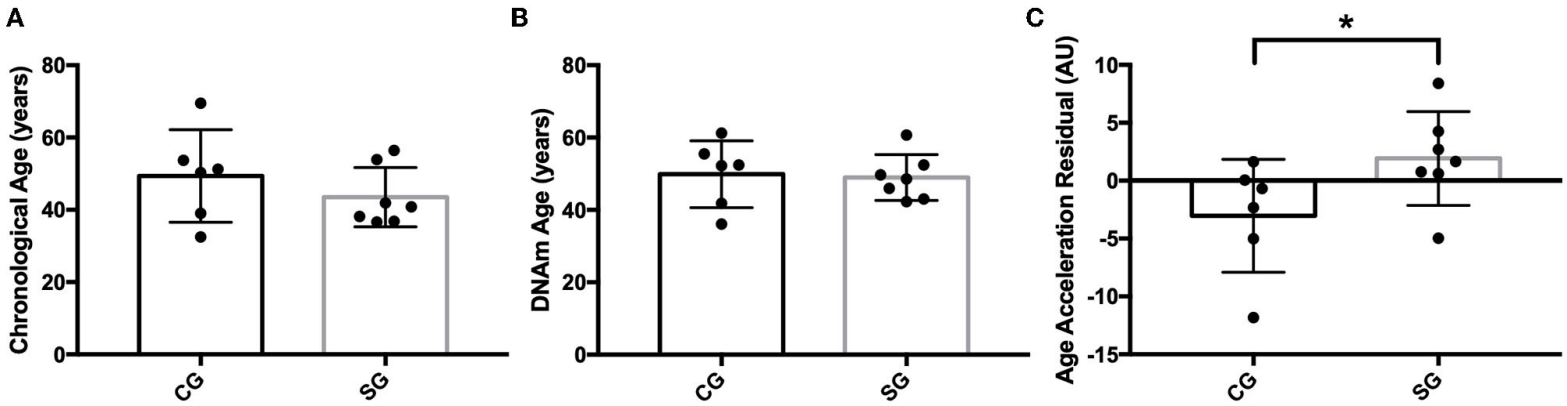
**(A)** Comparison of chronological Age (in years) between study group (SG) and control group (CG), **(B)** Comparison of DNA methylation age (DNAm) age (in years) between SG and CG, **(C)** Comparison of age acceleration residual (AAR) (in years) between SG and CG (p* = 0.036).

DNAm age was not different between SG and CG (DNAm age: 41.85 ± 13.04 vs. 40.80 ± 14.49, *p* = 0.86). However, AAR, which has more biological relevance as an indicator of an individual's rate of biological aging, was higher in the SG (Figure 1A). Furthermore, there was a negative correlation between AAR and the ZnS levels (Figure 1B), but AAR showed a positive correlation with BMI and WC (Figures 1C,D). When we reanalyzed the AAR by removing participants who were younger than 30 years old [SG: 43.51 ± 8.22 (*n* = 7), CG: 47.68 ± 9.87], there were no significant differences in the chronological age and DNAm age between the SG and CG (Figures 2A,B). Concomitantly, we observed a significant increase of AAR in SG participants from this sub-analysis (*p* = 0.036) (Figure 2C).

### Differentially Methylated Regions Calculation

The DMR analysis by the ChAMP package revealed that 30 DMRs were associated with ZnS levels, and 10 DMRs were associated with ZnDI (Table 2).

**Table 2:** Zinc-related differentially methylated regions (DMRs).

**DMRs**	**CpGs**	**CHR**	**Gene**	**DMRs**	**CpGs**	**CHR**	**Gene**
**ZnS levels**							
DMR1	7	6	*NFYA*	DMR 16	8	19	*SLC44A2*
DMR2	9	15	*UNC45A*	DMR_17	9	2	*HPCAL1*
DMR3	7	1	*PM20D1*	DMR 18	8	14	*MTA1*
DMR4	9	1	*OR2L13*	DMR_19	10	14	*METTL3*
DMR5	10	1	*CRYZ/TYW3*	DMR_20	10	2	*KIAA1841*
DMR6	15	11	*C11orf21*	DMR_21	7	19	*GNG7*
DMR7	9	17	*C17orf97*	DMR_22	8	16	*GCSH*
DMR8	12	11	*CSTF3*	DMR_23	9	11	*BUD13*
DMR9	11	19	*SUV420H2*	DMR_24	9	3	*FAM116A*
DMR10	9	19	*ZNF562*	DMR_25	9	7	*MRPS24*
DMR11	9	15	*KIAA0101*	DMR_26	8	13	*RASA3*
DMR12	9	4	*TBC1D14*	DMR_27	8	19	*DACT3*
DMR13	11	2	*OTX1*	DMR_28	8	4	*LOC93622*
DMR14	11	14	*ERO1L*	DMR_29	8	5	*FAM174A*
DMR15	8	5	*FLJ44606*	DMR_30	8	17	*NAT9*
**ZnID**							
DMR1	7	6	*NFYA*	DMR6	7	11	*SPON1*
DMR2	9	15	*UNC45A*	DMR7	9	7	*LRRC61*
DMR3	7	1	*PM20D1*	DMR8	14	5	*MIR886*
DMR4	19	6	*RNF39*	DMR9	8	2	*PAX8*
DMR5	9	17	*PLD6*	DMR10	8	4	*DDX60*

*DMRs, Differentially methylated regions; CHR, chromosome number; The DMRs were generated by the Bumphunter method incorporated in the Champ package and all association values had significantly False Discovery Rate (FDR < 0.05)*.

**Figure 3:**
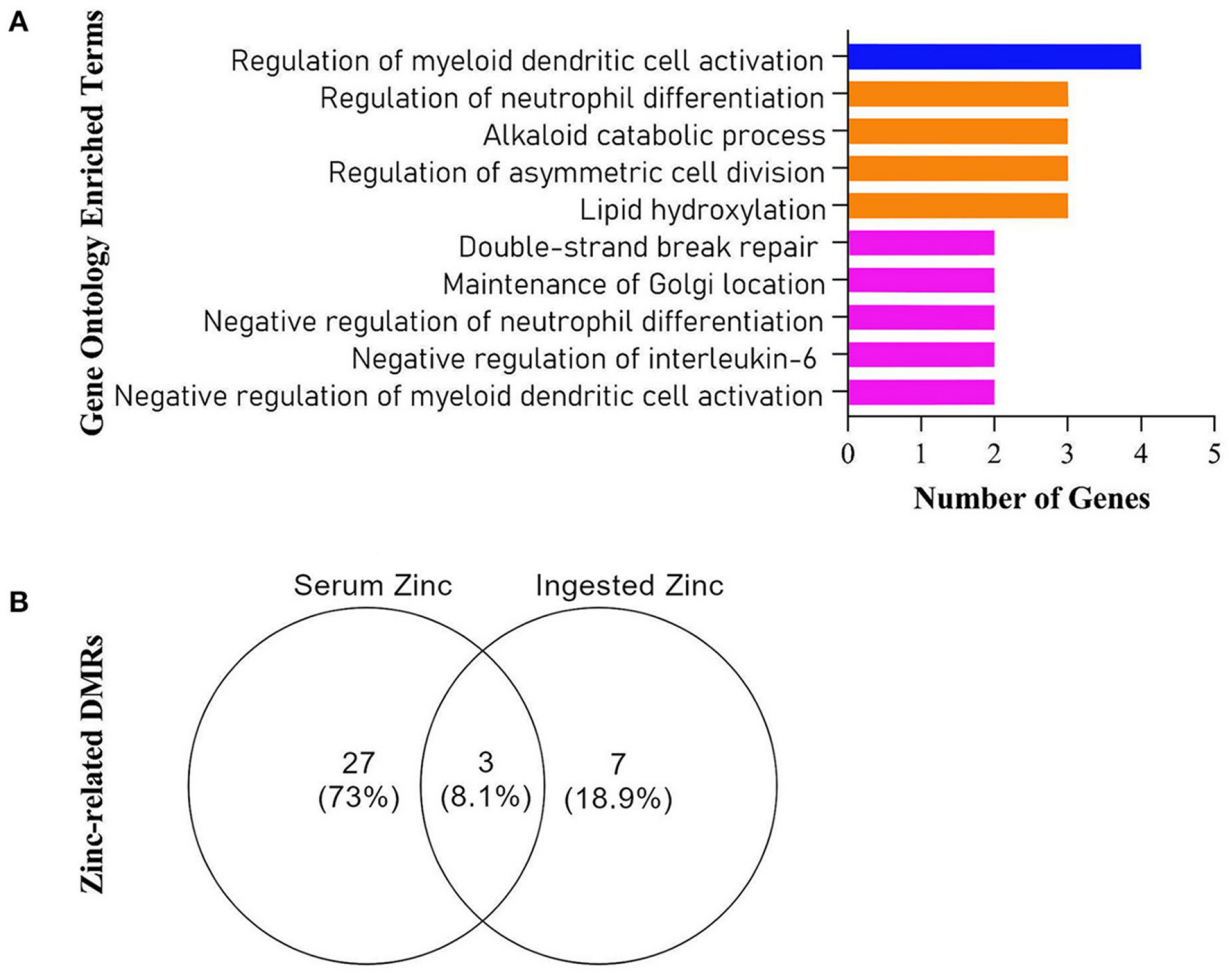
**(A)** Gene Ontology Enriched Terms performed on Webgestalt Web Tool, **(B)** Venn Diagram of all retrieved differentially methylated regions (DMRs) from the Champ algorithm.

The enrichment of all retrieved DMRs (Figure 3A) revealed targets involved with immune function (GO:0030886, GO:0045409, GO:0045659, GO:0045658, GO:0030885), DNA/cellular repair (GO:0051684, GO:0097680, GO:0009786), and lipid/protein metabolism (GO:0002933, GO:0009822). Three DMRs were common for both ZnDI and ZnS levels (Figure 3B). These targets were explored in depth in this article.

The common DMRs analyzed were enriched in the *NFYA, UNC45A*, and *PM20D1* genes, named DMR1, DMR2, and DMR3, respectively. No difference between groups for M value was detected for *NFYA* and *UNC45A* genes (Table 3). For the *PM20D1* gene, SG presented a lower M value compared to CG (-1.532 ± 1.369 vs. −0.353 ± 0.92, *p* = 0.031). From a site-specific perspective, six CpG sites' M values from the *PM20D1* gene were lower in the SG compared to the CG: cg17178900, cg07167872, cg11965913, cg14159672, cg14893161, cg24503407; *p* < 0.05. Table 3 also shows each group's beta value (β) and delta beta value (Δβ). Considering the SG as the reference, all the Δβ values from the *PM20D1* were negative, indicating that those sites were hypomethylated.

**Table 3:** M value mean comparison between study group (SG) and control group (CG) for each differentially methylated region (DMR) and its respective CpG sites, individually.

**ID sites**	**GENE (CHR)**	**SG**	**SG**	***p*** **-value**	**OG**	**CG**	**Δβ**
		**M value**	**M value**		**β**	**β**	
		**M ±SD**	**M ±SD**				
	***PM20D1*** **(1)**	−1.532 ± 1.369	−0.353 ± 0.921	**0.031**	0.303	0.452	−0.149
cg17178900	*PM20D1*	−1.020 ± 1.108	0.251 ± 0.893	**0.009**	0.348	0.543	−0.195
cg07157834	*PM20D1*	0.126 ± 0.985	0.792 ± 0.651	0.097	0.524	0.629	−0.105
cg07167872	*PM20D1*	−1.683 ± 1.478	0.399 ± 1.111	**0.035**	0.277	0.446	−0.169
cg11965913	*PM20D1*	−2.929 ± 1.933	−1.388 ± 1.230	**0.040**	0.174	0.305	−0.131
cg14159672	*PM20D1*	−1.487 ± 1.550	−0.213 ± 1.010	**0.036**	0.304	0.473	−0.169
cg14893161	*PM20D1*	−2.119 ± 1.304	−1.015 ± 0.821	**0.030**	0.219	0.343	−0.124
cg24503407	*PM20D1*	−1.649 ± 1.385	−0.501 ± 0.881	**0.034**	0.277	0.424	−0.147
	***NFYA*** **(6)**	3.642 ± 1.515	4.158 ± 0.525	0.5	0.916	0.937	−0.021
cg03644281	*NFYA*	4.589 ± 1.881	5.344 ± 0.778	0.36	0.949	0.972	−0.023
cg04346459	*NFYA*	3.686 ± 1.867	4.280 ± 0.751	0.778	0.915	0.945	−0.03
cg09580153	*NFYA*	4.213 ± 1.650	5.052 ± 0.785	0.481	0.944	0.967	−0.023
cg25110423	*NFYA*	2.956 ± 1.355	3.429 ± 0.584	0.725	0.885	0.91	−0.025
cg02167203	*NFYA*	3.578 ± 1.394	3.765 ± 0.333	0.526	0.917	0.93	−0.013
cg06671660	*NFYA*	2.880 ± 1.113	3.097 ± 0.443	0.778	0.884	0.892	−0.008
cg20398880	*NFYA*	3.681 ± 1.625	4.142 ± 0.554	0.833	0.921	0.943	−0.022
	***UNC45A*** **(15)**	−3.486 ± 0.759	−3.244 ± 0.696	0.455	0.096	0.109	−0.013
cg01351822	*UNC45A*	−3.436 ± 1.026	−3.226 ± 1.205	0.673	0.1	0.119	−0.019
cg08267442	*UNC45A*	−2.818 ± 0.499	−2.523 ± 0.456	0.175	0.129	0.152	−0.023
cg13778201	*UNC45A*	−2.384 ± 0.457	−2.232 ± 0.471	0.462	0.165	0.18	−0.015
cg03291024	*UNC45A*	−3.666 ± 0.728	−3.475 ± 0.660	0.535	0.08	0.089	−0.009
cg08551047	*UNC45A*	−3.611 ± 1.164	−3.237 ± 0.660	0.438	0.094	0.112	−0.018
cg08939371	*UNC45A*	−3.825 ± 0.864	−3.419 ± 0.605	0.225	0.075	0.091	−0.016
cg16414568	*UNC45A*	−3.651 ± 0.888	−3.339 ± 0.847	0.421	0.085	0.101	−0.016
cg18472881	*UNC45A*	−3.904 ± 0.941	−3.741 ± 0.710	0.656	0.074	0.076	−0.002
cg18724928	*UNC45A*	−4.079 ± 0.749	−4.002 ± 0.609	0.8	0.062	0.063	−0.001

*M ± SD, mean ± standard deviation; CG, control group; SG, study group; DMRs, Differentially methylated regions; CHR, chromosome number; t-test or Mann-Whitney, FDR p < 0.05. p-value < 0.05*.

**Figure 4:**
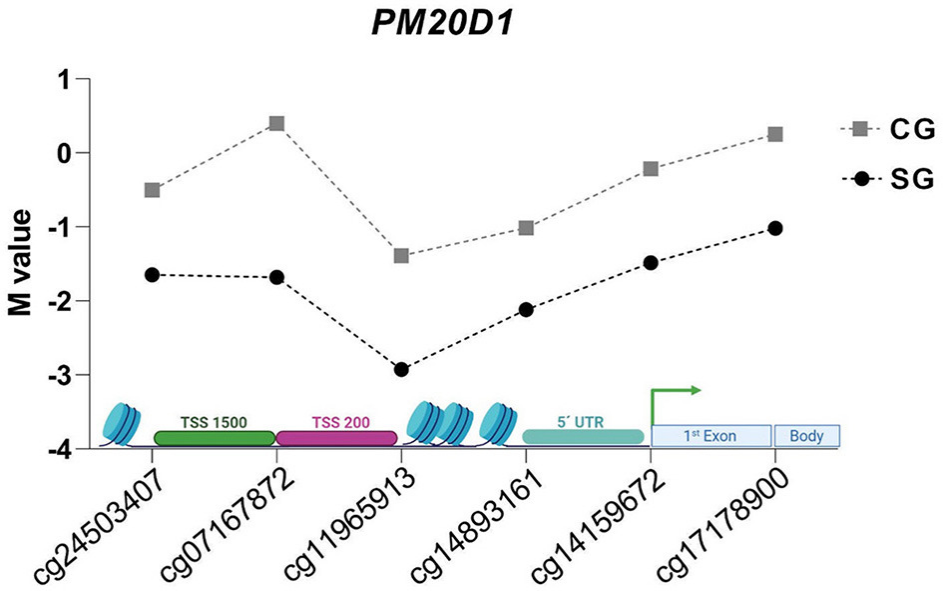
*M* values of evaluated CpG sites in DMR of *PM20D1* gene in SG e CG.

Interestingly, the majority (*n* = 6) of the CpG sites from the DMR3 (*PM20D1* genes) were located before the gene (TSS200, TSS1500, 5'UTR). One CpG was in the first exon, and one site was in the gene body (Figure 4). These associations reinforced that Zn might participate in gene control.

## Discussion

**Figure 5:**
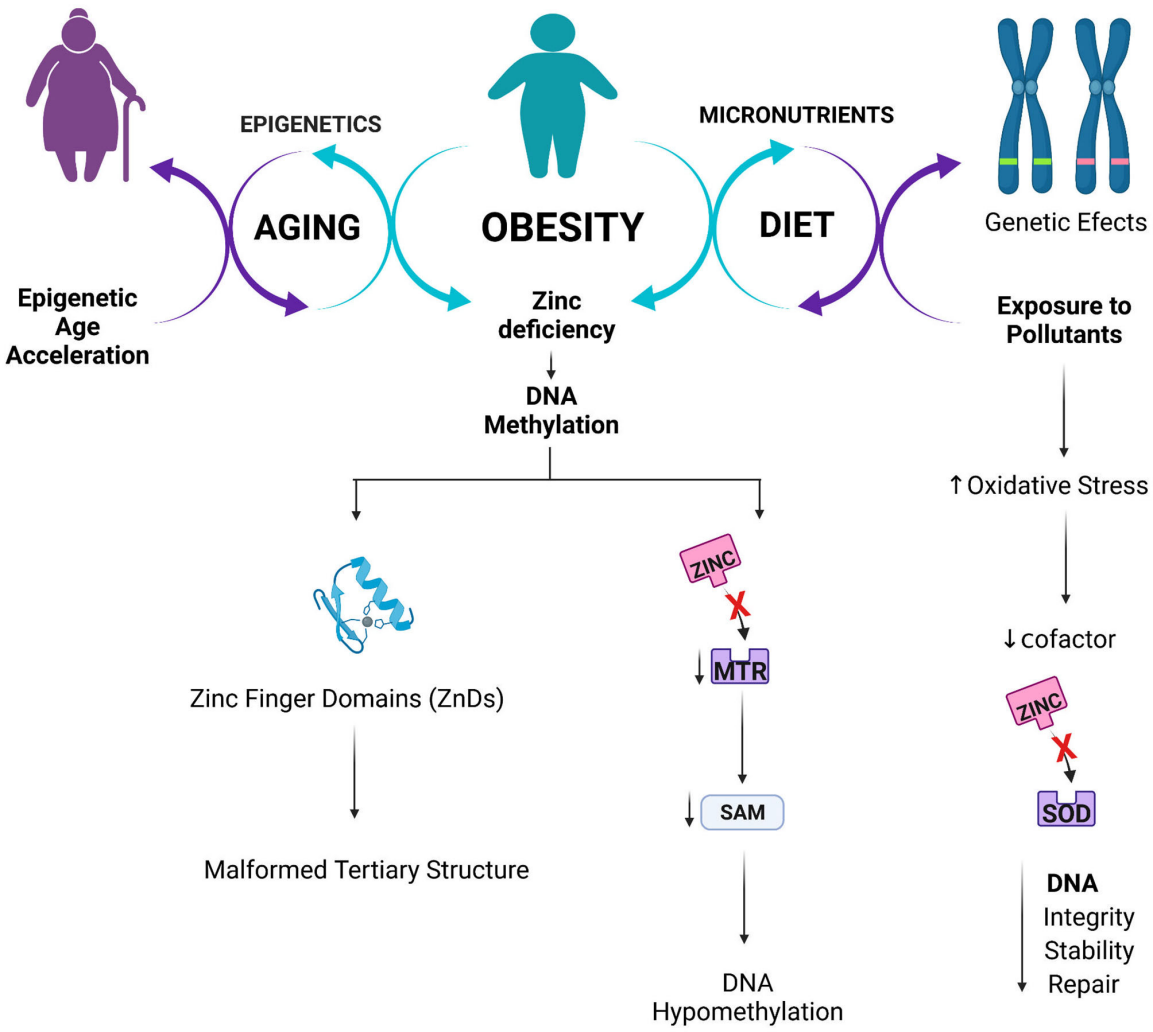
Proposed interlink between Zinc status, epigenetics and obesity.

The present study reinforced the concept that regulating human gene expression in obesity is a complex mechanism involving several aspects, including diet, genetic, epigenetic, and aging. Figure 5 summarizes the main mechanisms of action found in the present study.

Our study demonstrated an association between lower levels of Zn and obesity, which corroborated with previous data in the literature (30, 31). A randomized, double-blind clinical trial conducted with subjects with obesity compared the effects of Zn supplementation and placebo. The intervention group presented increased ZnS levels after Zn supplementation and decreased BMI compared to placebo (32). Furthermore, a cross-sectional study with children and adolescents found a negative association between ZnS levels and obesity (33).

Also, our results regarding Zn homeostasis in women with obesity suggested regulation by other mechanisms, besides ingestion, which corroborated with a previous study conducted in adults with dyslipidemia, in which they did not observe a correlation between ZnDI and ZnS levels (34). Several events may be related to lower ZnS levels in patients with obesity, especially modifications in Zn metabolism, including absorption, bioavailability, and urinary excretion (35, 36). Individuals with obesity could also have decreased expression of Zn transporters families, such as the Zrt-, Irt-like protein (ZIP) and the Zn transporter (ZnT), in the cell membrane, decreasing the Zn assimilation and consequently lower ZnS (36).

As observed in the present study, the dysregulation in Zn homeostasis can result in epigenetic alterations (36), reflecting in variations of the EAAR and DMRs and indicating that women with obesity age faster than those without obesity. Moreover, the negative correlation of ZnS with the epigenetic age acceleration residual (EAAR) was observed, suggesting that the higher the ZnS levels, the lower is the aging rate of the individual. Xiao et al. observed a negative correlation between age acceleration and Zn in a study that evaluated important factors for aging and longevity in a Chinese cohort (30).

Both ZnDI and ZnS levels were associated with DMRs in the DNA. Three DMRs were commonly associated with both ZnDI and ZnS levels, although the enrichment analysis indicated that only *PM20D1* differed between the SG and CG. A lower β mean value of *PM20D1* was detected in the SG compared to the CG, suggesting that SG has a hypomethylated phenotype for this target. The *PM20D1* enzyme synthesizes and hydrolyzes (bidirectional activity) the endogenous N-fatty acyl amino acid (NAAs). This protein is found in many tissues and can influence body energy expenditure (31). Indeed, Benson et al. (37) observed a lower percentage of methylated regions at *PM20D1* in the presence of *rs823080* polymorphism in *PM20D1* gene, increasing the expression of the gene and resulting in a lower NAA levels, higher BMI, and higher risk for diabetes mellitus type 2. The *PM20D1* gene is also important in regulating the PPARγ. PPARs are ligand-activated transcription factors, and PPARγ regulates fatty acid, glucose, and energy homeostasis (38). Studies have shown that Zn supplementation could increase PPARγ expression (39), since the PPAR-DNA binding seems to occur through ZnDs (16).

In this study, the DMR approach decreases the chance of negative random association compared to single CpG site's high reproducible technique. Furthermore, standardized arrays and bioinformatics analysis can make data more comparable through datasets. Data sharing and adoption of standardized bioinformatic methods are essential for the development of nutrigenomics and nutriepigenetics. Leukocytes are important components in obesity studies and may carry epigenetic marks due to the chronic low-grade inflammatory status (40). The mixed cell types could be considered a limiting factor, but this bias was alleviated by the cell types correction using Houseman's method (21). Previous studies have shown that Zn levels fluctuate widely in line with menstrual cycle (41, 42). However, despite the prevalence of female gender in this study, the Zn status according to the menstrual cycle was a limitation in this cohort. As far as we know, this is the first study that evaluated the association of ZnDI and ZnS levels and human leukocytes epigenome in individuals with obesity.

## Conclusion

The present study evidences the influence of Zn in the modulation of DNA methylation patterns. Obesity parameters are related to Zn deficiency and epigenetic age acceleration. Zn-associated DMRs may exert downstream effects on inflammation, macronutrient metabolism, and DNA/cellular process repair. The hypomethylation of the *PM20D1* gene could indicate the interconnection between DNA methylation and nutritional status. Thus, we emphasized the importance of monitoring Zn, not only in the serum measurement but also the ingestion during clinical treatments and follow-ups, to avoid Zn deficiency and consequently to decrease the risks for the development of chronic disease.

## Data Availability Statement

The datasets presented in this study can be found in online repositories. The names of the repository/repositories and accession number(s) can be found at: Gene Expression Omnibus, GSE193836. https://www.ncbi.nlm.nih.gov/geo/query/acc.cgi?acc=GSE193836.

## Ethics Statement

The studies involving human participants were reviewed and approved by Research Ethics Committee of the Ribeirão Preto Medical School University Hospital of University of São Paulo (HCRP-USP) CAAE license: 14275319.7.0000.5440. The patients/participants provided their written informed consent to participate in this study.

## Author Contributions

NN and MB conceived and designed the study. CN, MP, and FB provided laboratory support, supervised experiments, and got financial support. NN and MP collected clinical data. NN, MP, LW, VS, DM, and WS performed experiments. GR, NN, MB, VP, and JP performed data analysis. MB, NN, GR, and LW drafted the manuscript. GR curated the data to make it public. All authors participated in the manuscript review and approved the final manuscript.

## Funding

Consumables: São Paulo Research Foundation (FAPESP) (#2018/24069-3) and National Council for Scientific and Technological Development (CNPq: #408292/2018-0). Personal funding: (FAPESP: #2014/16740-6 and #2020/08687-9) and Academic Excellence Program from Coordination for higher Education Staff Development (CAPES: 88882.180020/2018-01). Moreover, this work was supported by the São José do Rio Preto Medical School (FAMERP) and the Foundation to Support Teaching, Research and Assistance of the Clinical Hospital, Faculty of Medicine of Ribeirao Preto, University of São Paulo (FAEPA).

## Conflict of Interest

The authors declare that the research was conducted in the absence of any commercial or financial relationships that could be construed as a potential conflict of interest.

## Publisher's Note

All claims expressed in this article are solely those of the authors and do not necessarily represent those of their affiliated organizations, or those of the publisher, the editors and the reviewers. Any product that may be evaluated in this article, or claim that may be made by its manufacturer, is not guaranteed or endorsed by the publisher.
