# Corrigendum: Novel Zinc-Related Differentially Methylated Regions in Leukocytes of Women With and Without Obesity

**DOI:** 10.3389/fnut.2022.911493

**Published:** 2022-04-28

**Authors:** Natália Yumi Noronha, Mariana Barato, Chanachai Sae-Lee, Marcela Augusta de Souza Pinhel, Lígia Moriguchi Watanabe, Vanessa Aparecida Batista Pereira, Guilherme da Silva Rodrigues, Déborah Araújo Morais, Wellington Tavares de Sousa, Vanessa Cristina de Oliveira Souza, Jessica Rodrigues Plaça, Wilson Salgado, Fernando Barbosa, Torsten Plösch, Carla Barbosa Nonino

**Affiliations:** ^1^Department of Internal Medicine, Ribeirao Preto Medical School, University of São Paulo, São Paulo, Brazil; ^2^Department of Molecular Biology, São José do Rio Preto Medical School, São Paulo, Brazil; ^3^Research Division, Faculty of Medicine, Siriraj Hospital, Mahidol University, Bangkok, Thailand; ^4^Department of Health Sciences, Ribeirão Preto Medical School, University of São Paulo, São Paulo, Brazil; ^5^Department of Clinical Analysis, Toxicology and Food Sciences, School of Pharmaceutical Sciences of Ribeirão Preto, University of São Paulo, São Paulo, Brazil; ^6^National Institute of Science and Technology in Stem Cell and Cell Therapy and Center for Cell-Based Therapy, São Paulo, Brazil; ^7^Department of Surgery and Anatomy, Ribeirao Preto Medical School, São Paulo, Brazil; ^8^Department of Obstetrics and Gynecology, University Medical Center Groningen, University of Groningen, Groningen, Netherlands

**Keywords:** zinc deficiency, DNA methylation, age acceleration, epigenetic markers, *PM20D1*

In the original article, there was a mistake in Figures 1, 3, and 4. The figures originally had a white background, however, in published article the background is black. The updated figures are shown below.

**Figure 1:**
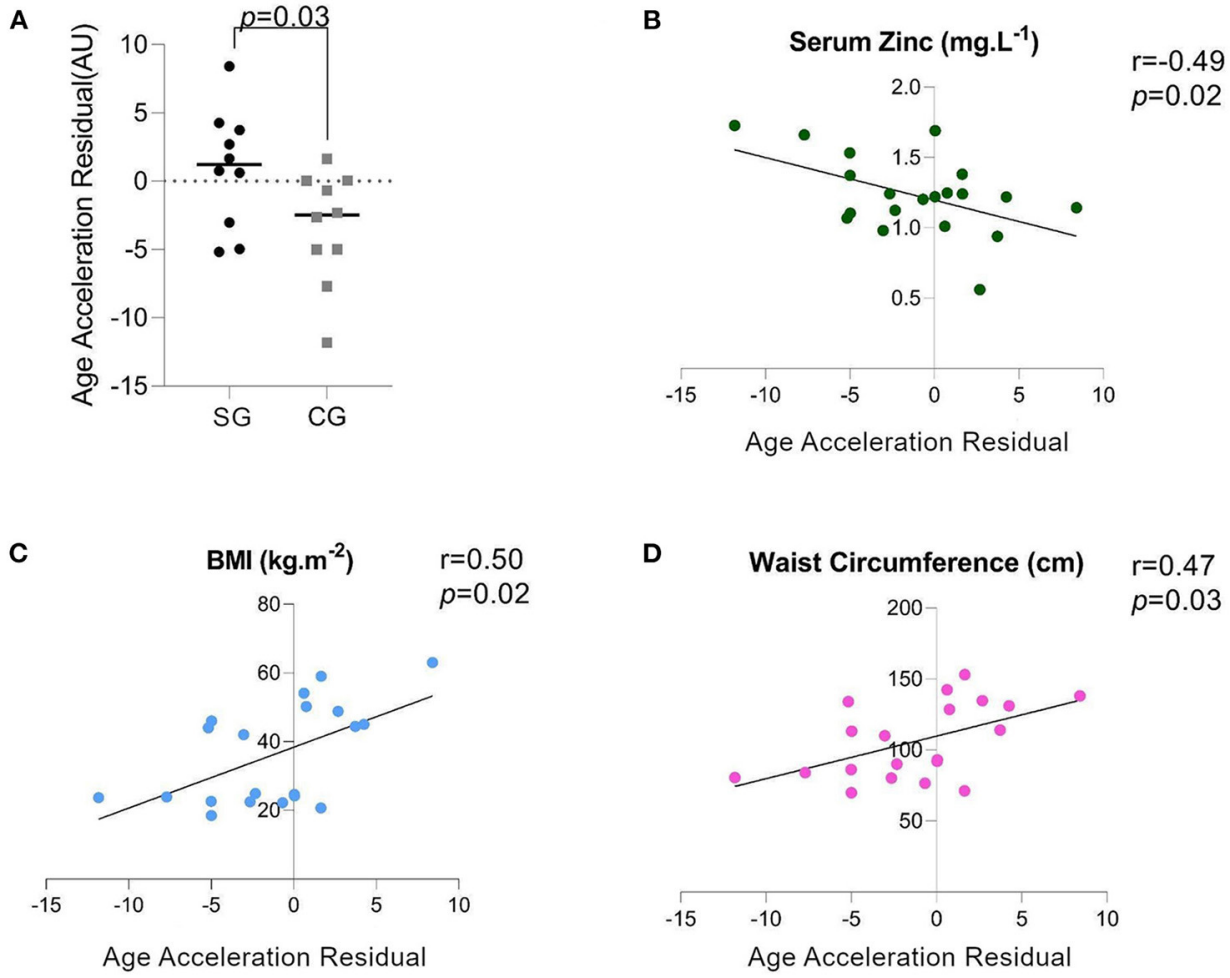
**(A)** AAR: Age Acceleration Residual (measured in arbitrary units), **(B)** Spearman's Correlation of the serum zinc (ZnS) levels and the AAR, **(C)** Spearman's Correlation of the BMI and AAR, **(D)** Spearman's Correlation of the waist circumference (WC) and AAR.

**Figure 3:**
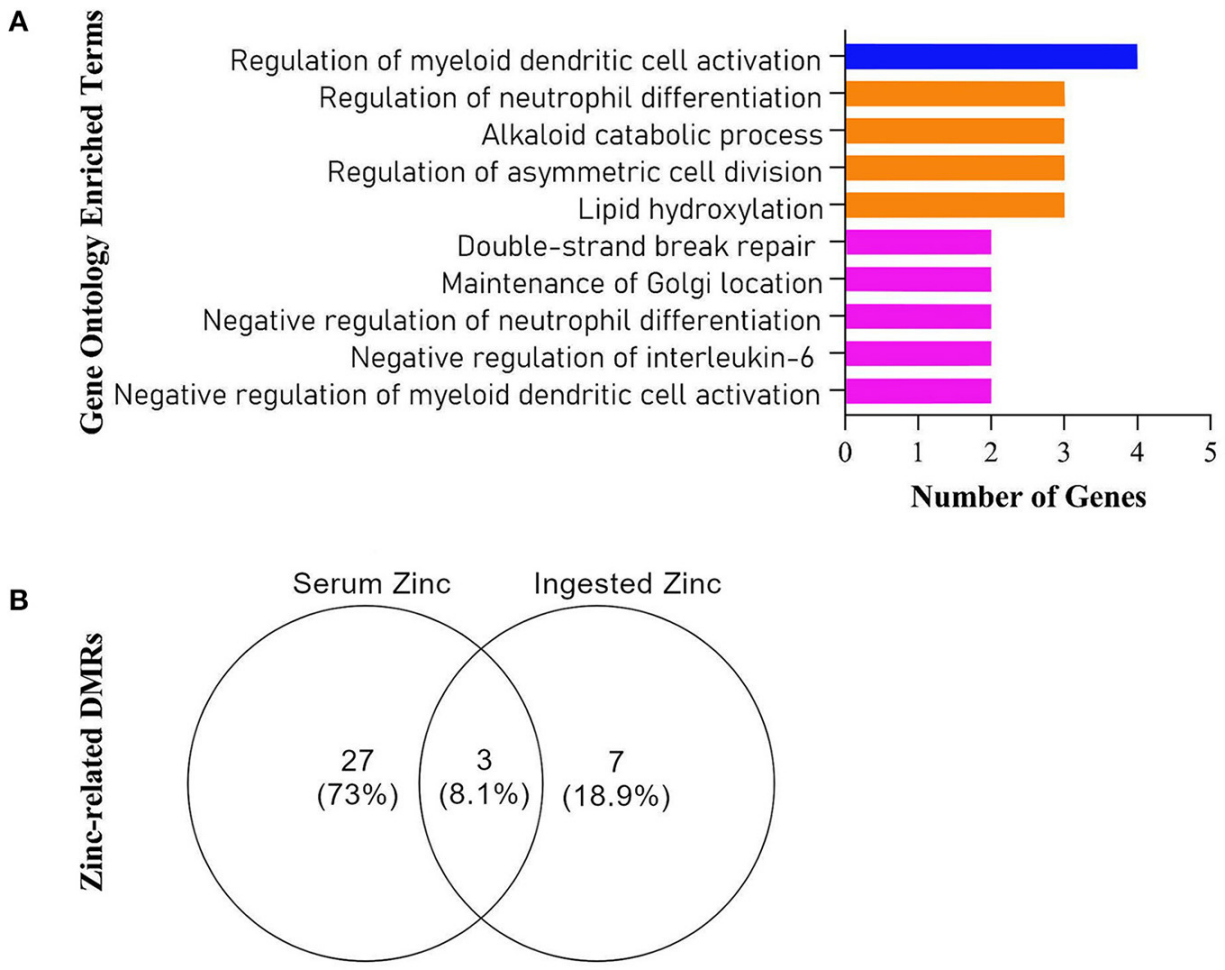
**(A)** Gene Ontology Enriched Terms performed on Webgestalt Web Tool, **(B)** Venn Diagram of all retrieved differentially methylated regions (DMRs) from the Champ algorithm.

**Figure 4:**
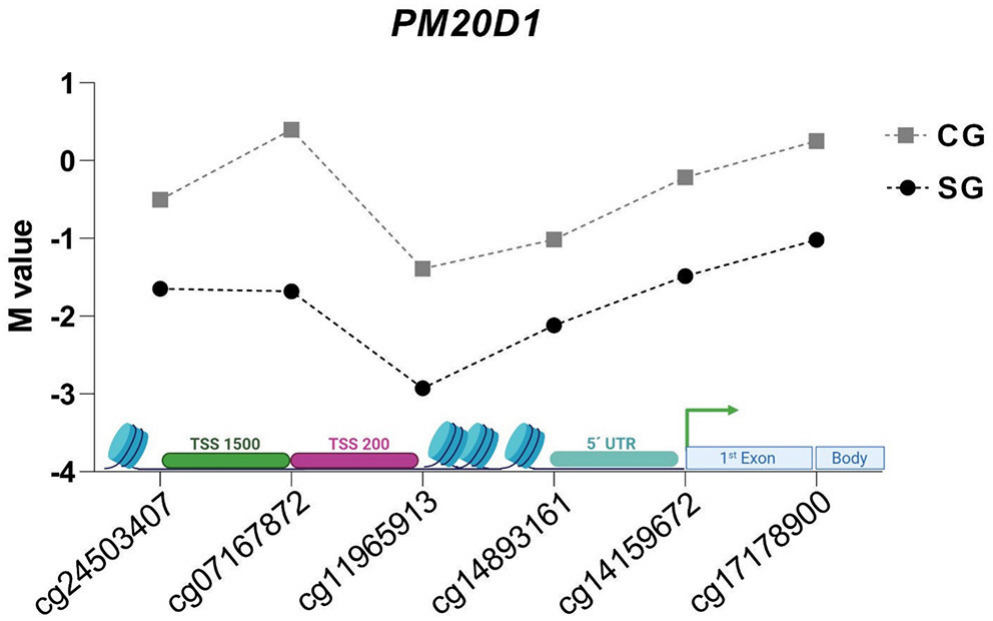
*M* values of evaluated CpG sites in DMR of *PM20D1* gene in SG e CG.

The authors apologize for this error and state that this does not change the scientific conclusions of the article in any way. The original article has been updated.

## Publisher's Note

All claims expressed in this article are solely those of the authors and do not necessarily represent those of their affiliated organizations, or those of the publisher, the editors and the reviewers. Any product that may be evaluated in this article, or claim that may be made by its manufacturer, is not guaranteed or endorsed by the publisher.

